# ﻿Phylogeny of the European *Collema* species (Peltigerales, Lecanoromycetes)

**DOI:** 10.3897/mycokeys.115.144718

**Published:** 2025-03-18

**Authors:** Alica Košuthová, Fredrik Jonsson, Ulrika Nordin, Mats Wedin

**Affiliations:** 1 Department of Botany, Swedish Museum of Natural History, P.O. Box 50007, SE-104 05 Stockholm, Sweden Department of Botany, Swedish Museum of Natural History Stockholm Sweden; 2 Alsens-Ede 227, SE-835 96 Trångsviken, Sweden Unaffiliated Trångsviken Sweden

**Keywords:** Ascomycota, Collemataceae, cyanolichens, integrative taxonomy, systematics

## Abstract

The phylogenetic relationships and morphological diversity within European *Collema* s. str. species were investigated. A total of 104 new sequences (four molecular markers; mtSSU, b-tub, MCM7, and RPB2 genes) from 28 specimens were generated, and analysed and used for multi-locus phylogenetic analyses. Our results suggest that *Collema* is only monophyletic if *Collemaglebulentum* is considered part of *Leptogiums. str.* where it originally was described. This is supported by its paraplectenchymatous thallus. Degelius´ informal *Collema* “*Flaccidum*”- and “*Nigrescens*”-groups are not natural, as the “*Flaccidum*”-group is nested within the “*Nigrescens*”-group. Based on our findings, seven currently accepted *Collema* occur in Europe: *C.flaccidum*, *C.subflaccidum*, *C.curtisporum*, *C.furfuraceum*, *C.nigrescens*, *C.ryssoleum*, and *C.subnigrescens*. *Collemafurfuraceum* is further non-monophyletic, suggesting a need for taxonomic revision.

## ﻿Introduction

The generic delimitation of Collemataceae s. str. was for a long time unnatural, relying primarily on a single trait: the presence of a cellular cortex in *Leptogium* or its absence in *Collema* (Degelius 1954, 1974; Jørgensen 2007). Degelius (1954) had, however, already questioned whether *Collema* and *Leptogium* represented natural, monophyletic groupings. Subsequent molecular studies (Wiklund and Wedin 2003; Miadlikowska and Lutzoni 2004; Miadlikowska et al. 2014) supported this, revealing that the gelatinous genera with simple spores, traditionally assigned to Collemataceae, actually belonged to other families (Wedin et al. 2009; Otálora et al. 2010; Ekman et al. 2014; Weerakoon et al. 2018). It was not until the comprehensive analysis by Otálora et al. (2013a, 2013b) that a revised generic classification of Collemataceae s. str. was proposed. Their work confirmed the non-monophyletic nature of *Collema* and *Leptogium*, proposing the recognition of ten distinct monophyletic groups as separate genera. This revision retained the names *Collema* and *Leptogium* in restricted senses and reintroduced six older generic names (*Blennothallia*, *Enchylium*, *Lathagrium*, *Pseudoleptogium*, *Rostania*, and *Scytinium*), while also establishing two new genera, *Callome* and *Paracollema*.

The European species of *Collema* in the sense of Otálora et al. (2013b), the focus of the present study, correspond to Degelius’ “*Flaccidum*” and “*Nigrescens*”-groups (Degelius 1954). These groups are relatively small, with three species currently recognized in the “*Flaccidum*”-group and five in the “*Nigrescens*”-group. The two groups are closely related, sharing several characteristics, including their occurrence in sheltered, humid environments, and the two groups contain both corticolous and saxicolous species. Species within these groups are large in size, typically measuring about 2.0–8.0 cm in diameter, though some individuals may reach up to 15 cm. Their thalli are thin to moderately thin (50–150 µm), lacking both a paraplectenchymatous medulla, and a true cortex. The lobes are generally broad and rounded, measuring approximately 5.0–12.0 mm in diameter. The spores of both groups are ranging from 25–35 × 6–7 μm to 40–90 × 3–6 μm, and are narrowly ellipsoid to fusiform with transverse septation. Spores in the “*Nigrescens*”-group show greater polymorphism than those in the “*Flaccidum*”-group. A distinguishing feature between the two groups is that species in the “*Flaccidum*”-group lack pustules and ridges, whereas those are present in species of the “*Nigrescens*”-group. Both groups are distributed across Europe, with their distribution range and selected morphological features summarized in Table 1. Degelius (1954, 1974) accepted three European species in the “*Flaccidum*”-group: *Collemaflaccidum* (Ach.) Ach., *C.glebulentum* (Cromb.) Degel., and *C.subflaccidum* Degel. previously known as *C.subfurvum* (Müll. Arg.) Degel. The five European species in the “*Nigrescens*”-groups include *C.curtisporum* Degel., *C.furfuraceum* (Arn.) DR. em. Degel., *C.nigrescens* (Huds.) DC., *C.ryssoleum* (Tuck.) Schneid., and *C.subnigrescens* Degel.

**Table 1.** Characteristic morphological features of European *Collema* species summarised according to Degelius (1954, 1974). Characters unique for the species in the “*Flaccidum*” and “*Nigrescens*” -groups are in bold.

Group	Species	Morphology														
	(*Collema*)	Pustules/ridges	Thallus (cm)	Thallus thickness (µm)	Thallus colour	Lobes (cm)	Isidia	Isidia width (mm)	Isidia length (mm)	Apothecium (mm)	Apothecium disc	Spore measurements (µm)	Spore shape	Spore septas	Habitat	Distribution
*Flaccidum*	*flaccidum*	N	to 6	70–170	olive-green-blackish	0.5–1.5(3)	**squamiform** (juv. globular)	0.2–0.5	min. 0.2–0.5	rare, to 1.5	pale red to dark red, sometimes pruinose	(20)26–34(45) × 6–6.5(8.5)	fusiform with acute ends	4 to 6	saxi- and corti-colous	wide, suboceanic
	*glebulentum*	N	(1)3–6	(50)65–135(200)	light to dark olive-green-blackish	to 1	teretiform, coralloid, **capitate**	0.1	1.5	N	N	N	N	N	**saxicolous**	**arctic-alpine**
	*subflaccidum*	N	to 6	80–130	olive-green-blackish	0.5–1.5(3)	**globular**, old teretiform & coralloid	0.05–0.1	min. 0.3	rare, to 1.5	pale red to dark red, epruinose	42–60(65) × (3)4.5–6.5(10.5)	broadly to narrowly fusiform, acicular	(4)6–8	corti- and saxi-colous	**oceanic**
*Nigrescens*	*curtisporum*	Y	to 3(4)	65–106	dark olive-green-blackish	0.5–1	N	N	N	0.5–1.5	pale-red-dark red-brown-blackish, epruinose	**(18)26–34(40) × 3–4.5**	bacillar, straight or **curved, obtuse ends**	4(5–6)	corticolous	**boreal**
	*furfuraceum*	Y	3–6(10)	(50)60–105	dark olive-green-blackish	0.5–1	**teretiform, coralloid, branched** (juv. globular)	**0.05–0.15**(0.2)	**0.3**	very rare, 0.5–1.5	pale-red-dark red-brown-blackish, epruinose	40–80 × 3–6(8.5)	bacillar-narrowly fusiform with acute end, one end thicker and usually curved	5 to 6	corticolous	wide, suboceanic
	*nigrescens*	Y	to 10	**(60)90–150**	dark olive-green, brownish-blackish	0.5–1	**globular**	**0.2**	**0.2**	often, 0.6–1	pale-red-dark red-brown-blackish, epruinose	**50–90(112) × 3–4.5**	**acicular to bacillar**	**(5)6–13**	corticolous	wide, suboceanic
	*ryssoleum*	Y	to 22	50–85	dark olive-green-blackish	0.5–1.5	N	N	N	0.6–1(1.5)	dark red-blackish, epruinose	**(22)26–40(47) × (4.5)5–8.5(10.5)**	ellipsoid or fusiform, **broader in middle**, with acute ends	4 to 6	**saxicolous**	**temperate - mediterranean zone**
	*subnigrescens*	Y	5–10(20)	60–100	dark olive-green-blackish	0.5–1.5	N	N	N	often, 1–1.5(2)	pale-red-dark red-brown-blackish, epruinose	(34)40–75 × 6–6.5(7)	narrowly fusiform with acute end, one end thicker and usually curved	5 to 6	corticolous	wide, suboceanic

Otálora et al. (2013a, 2013b) included only four species (*C.curtisporum*, *C.flaccidum*, *C.furfuraceum* and *C.nigrescens*) of the European *Collema* s. str. in their phylogenies, with Miadlikowska et al. (2014) also including *C.subnigrescens*. As a result, the taxonomic positions of five out of eight species within this group have been tested using molecular methods, while the remaining three species have not yet been examined. As there is a substantial variation in shape and size of the thallus, lobes, apothecia, ascospores, and isidia among them, and as several former *Collema* species have been shown to belong elsewhere, the delimitation of the genus needs investigation. Here, we will test the current delimitation of *Collema* in Europe and propose a phylogenetic hypothesis of known species. Finally, we will note and comment on any indication of species non-monophyly in this genus.

## ﻿Material and methods

### ﻿Specimen selection and morphological observations

We sampled 28 specimens, representing species of the European “*Flaccidum*” *and* “*Nigrescens*”-groups from Scandinavia and including some extra-Scandinavian material for comparison. The collections resulting from our own recent fieldwork are deposited in the herbarium S, with additional material studied from GZU, hb. Malíček, TBS, UPS and S. Collections and the sequences used are summarized in Table 2. Herbarium acronyms follow Thiers (2018).

**Table 2.** Sequences utilized in this study. Newly produced sequences in bold with herbarium vouchers of the specimens given, remaining sequences downloaded from GenBank. For specimens of *Collemaglebulentum*, origin of both, state and provinces are given.

Specimen	DNA-voucher	Year	Locality & Herbarium voucher	mtSSU	BT	MCM7	RPB2 5-7	RPB2 7-11
*Leptogiumbyssinum*			Norway: *Westberg* (S)	KT240180		KT240183		
*Leptogiumterrenum*			Portugal: *van den Boom 41781* (hb. van den Boom)	KT240181		KT240184		
*Collemaglebulentum*	AL561	2015	Sweden, Pite Lappmark (Arjeplog): *Westberg 15-254* (S-F277955)	**PQ932211**	**PV021123**		**PV021148**	
*Collemaglebulentum*	AL560	1988	Sweden, Åsele Lappmark (Vilhelmina): *Thor 7711* (S-L49768)	**PQ932210**				
*Collemaglebulentum*	AL674	2018	Sweden, Värmland (Säffle-Lurö): *Košuthová et al. 401* (S-F492346)	**PQ932212**	**PV021124**		**PV021149**	
*Collemaglebulentum*	AL366	2017	Sweden, Närke (Örebro): *Berglund* (S-F492347)	**PQ932209**	**PV021122**			
*Leptogiumazureum*			Chile: *Cornejo 26507* (MA)	JX992939	KC119021	JX993002		
*Leptogiumdenticulatum*		2010	Argentina: *Wedin 8690* (S-F332474)	JX992947	KC119025	JX993012	**PV021147**	
*Collemasubconveniens*		2010	New Zealand: *Wedin 9225* (S-F335747)	JX992937	KC119019	JX992996	**PV021150**	
*Collemaleptaleum*			Argentina: *Wedin 8822* (S)	JX992928	KC119038	JX992986		
*Collemaflaccidum*	AL540	2016	Sweden: *Westberg et al. 244* (UPS-L872188)	**PQ932216**	**PV021129**	**PV021173**	**PV021154**	
*Collemaflaccidum*	AL496	2017	Sweden: *Odelvik 17-523* (S-F317108)	**PQ932215**	**PV021128**	**PV021172**	**PV021153**	**PV021189**
*Collemaflaccidum*	AL531_AL494	2018	Slovakia: *Košuthová et al. 601* (S-F492348)	**PQ932217**	**PV021130**	**PV021174**	**PV021155**	**PV021190**
*Collemasubflaccidum*	AL649	2016	Russia: *Malíček et al. 10619* (S-F492349 & dupl. herb. Malíček)	**PQ932214**	**PV021132**		**PV021152**	
*Collemasubflaccidum*	AL495	2018	Norway: *Aptroot 76306* (S-F492350)	**PQ932213**	**PV021131**		**PV021151**	**PV021188**
*Collemanigrescens*	AL511	2018	Spain: *Westberg* (UPS-L934034)	**PQ932220**	**PV021127**		**PV021158**	
*Collemanigrescens*	AL493	2018	Slovakia: *Košuthová et al. 600* (S-F492351)	**PQ932219**	**PV021126**		**PV021157**	
*Collemanigrescens*	AL603	2018	Sweden : *Košuthová & Arvidsson 571* (S-F492352)	**PQ932218**	**PV021125**	**PV021175**	**PV021156**	
*Collemacurtisporum*	AL411	2017	Sweden: *Jonsson & U.Nordin FU6546* (S-F492353)	**PQ932221**	**PV021133**		**PV021159**	**PV021191**
*Collemacurtisporum*	AL568	1994	Sweden: *Hermansson 4603* (UPS-L111603)	**PQ932222**		**PV021176**	**PV021160**	
*Collemafurfuraceum*	AL668_AL721	2002	Sweden: *Jonsson 2254* (S-F492354)	**PQ932223**		**PV021177**	**PV021161**	**PV021192**
*Collemafurfuraceum*	AL640_AL720	1998	Sweden: *Bergsten* (S-F492355)	**PQ932224**		**PV021178**	**PV021162**	**PV021193**
*Collemaryssoleum*	AL518	2001	Italy: *Trietach* (TSB-35166)	**PQ932231**	**PV021135**	**PV021184**	**PV021168**	**PV021196**
*Collemaryssoleum*	AL534_AL513	1974	Spain: *Tibell 5610* (UPS-L933969)	**PQ932232**	**PV021136**			
*Collemaryssoleum*	AL566	1994	Madeira: *Nordin 3524* (UPS-L178905)	**PQ932233**	**PV021134**			
*Collemasubnigrescens*	AL407	2017	Sweden: *Jonsson & U.Nordin FU6531* (S-F492356)	**PQ932227**	**PV021140**	**PV021180**	**PV021165**	
*Collemasubnigrescens*	AL500	2006	Greece: *Spribille 19637* (GZU66-201)	**PQ932226**	**PV021141**		**PV021164**	
*Collemasubnigrescens*	AL570	2004	Estonia: *Odelvik 0485* (S-F57732)	**PQ932228**	**PV021142**	**PV021181**	**PV021166**	**PV021195**
*Collemasubnigrescens*	AL344	2017	Sweden: *Berglund* (S-F492357)	**PQ932225**	**PV021139**	**PV021179**	**PV021163**	**PV021194**
*Collemafurfuraceum*	AL644	2018	Scotland: *Malíček 12545* (herb. Malíček)	**PQ932230**	**PV021143**	**PV021183**		
*Collemafurfuraceum*	AL666	2012	Italy: *Jonsson A2* (S-F492358)	**PQ932229**		**PV021182**	**PV021167**	
*Collemafurfuraceum*	AL712	2022	Sweden: *Westberg* (UPS-L1049804)	**PQ932235**	**PV021138**	**PV021186**	**PV021170**	
*Collemafurfuraceum*	AL536	2018	Spain: *Westberg* (UPS-L934040)	**PQ932234**	**PV021137**	**PV021185**	**PV021169**	**PV021197**
*Collemafurfuraceum*	AL665	2012	France: *Jonsson A3* (S-F492359)	**PQ932236**	**PV021144**	**PV021187**	**PV021171**	
*Paracollema italicum3*			Croatia: *Nordin 2763* (UPS)	JX992926		JX992985		
*Paracollema italicum1*			Croatia: *Nordin 2708* (UPS)	JX992925	KC119015	JX992984		
*Callome multipartita1*			Norway: *Haugan 7015* (O)	GQ259019				
*Callome multipartita2*	AL419	2009	Austria: *Hafellner 74818* (GZU-18–2009)	MK445271	MK451935		**PV021146**	
*Enchyliumbachmanianum*	MWE111	1985	Sweden: *Nordin 1521* (UPS-L133627)	JX992914	MK451936	JX992974	**PV021145**	

Anatomical features were studied using a light microscope on thin sections cut with a razor blade or squash preparations mounted in water. Measurements of mature spores were taken outside of the asci under × 1000 magnification using oil immersion with a precision of 0.5 μm, or from calibrated digital photographs using NIS-Elements (Nikon, Japan) with a precision of 0.1 μm. Spore measurements are presented in the format: (minimum value observed–) range including 80% of the observed values (–maximum value observed), with the mean of all observed values in the center and italicized (Table 1). Full lists of specimens examined in this study with DNA voucher codes and GenBank Accession numbers for newly generated sequences are given in Table 2.

### ﻿DNA extraction, amplification and sequencing

Two apothecia with surrounding thalline parts, or a thallus fragment in the case of sterile samples, were selected for extraction. We extracted total DNA using the Plant DNA Mini Kit (Qiagen, Hilden, Germany) following the manufacturer’s instructions except in order to increase the concentration of DNA, we used half the amount of Elution buffer in the last step. We amplified one mitochondrial ribosomal and three nuclear protein-coding genes. Approximately 0.8 kb of the small subunit of the mitochondrial rDNA (mtSSU) was amplified using the primers mrSSU1 and mrSSU3R (Zoller et al. 1999). Approximately 0.6 kb of Beta-tubulin (b-tub) was amplified and sequenced using the primers Bt3-LM5 and Bt10-LM3 (Myllys et al. 2001) and BetaCollF and BetaCollR (Otálora et al. 2013a). The PCR primers were used in the following combinations: Bt3-LM5 and Bt10-LM3, BetaCollF and BetaCollR, Bt3-LM5 and BetaCollR, BetaCollF and Bt10-LM3 (the best working combination), BetaCollF and BetaColl-intR, and BetaColl-intF2 and Bt10LM3. About 0.6 kb of mini–chromosome maintenance complex component 7 DNA replication licensing factor (MCM7) was amplified and sequenced using the primers Mcm7-709for and Mcm7-1348rev (Schmitt et al. 2009). The locus RNA polymerase II protein coding gene (RPB2, ~2 kb) was amplified as two parts: ~ 1 kb for RPB2 5–7 and ~ 1 kb for RPB2 7–11. Amplification was performed using the primers fRPB2-5F, fRPB2-7cF, fRPB2-7cR and fRPB2-11aR (Liu et al. 1999), which were also used as sequencing primers. PCR amplifications were performed using Illustra™ Hot Start PCR beads, according to manufacturer’s instructions. PCR reactions were performed using the same settings as in previous studies (Košuthová et al. 2016, 2019; Otálora et al. 2013a).

### ﻿Sequence editing, alignment and phylogenetic analyses

The generated sequences were assembled and edited using Geneious version R8 (http://www.geneious.com, Kearse et al. 2012). All edited sequences underwent initial identity verification through BLAST searches (Zhang et al. 2000). The alignment of these sequences was performed using the MUSCLE algorithm (Edgar 2004) in AliView 1.09 (Larsson 2014). An intron in the b-tub was excluded. Indels in the mtSSU were not deleted, allowing smaller gap positions within the final block. The five genetic regions defined above were analysed separately using Maximum Likelihood (ML). As no significant incongruence was detected, the alignments were concatenated. The final alignment has been deposited in TREEBASE (http://www.treebase.org) with accession number (http://purl.org/phylo/treebase/phylows/study/TB:S31975). After concatenation, we inferred phylogenetic relationships using ML with the same settings used as in the individual gene analyses utilizing RAxML. Likelihood and ML bootstrapping were executed through RAxML 8 (Stamatakis 2014) implementing a general time reversible (GTR) model of nucleotide substitution with gamma distributed rate heterogeneity (GTRGAMMA). One thousand bootstrap (BS) replicates were completed using the non-parametric BS algorithm of RAxML–HPC v.8 on XSEDE using the CIPRES Web Portal (Miller et al. 2010).

**Figure 1.**
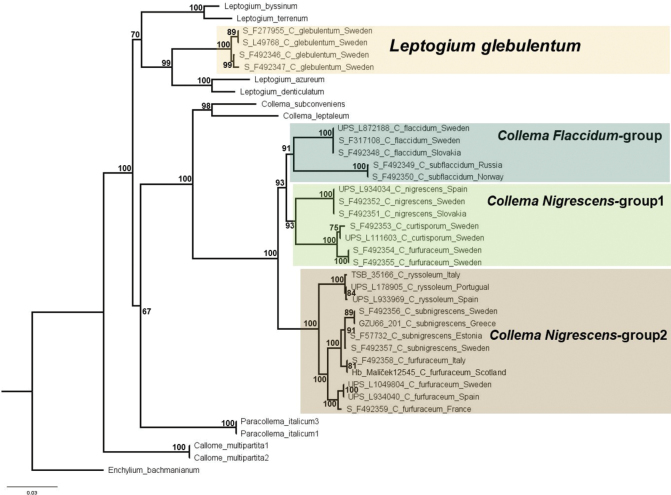
The most likely tree from RAxML analysis based on 4140 aligned characters of mtSSU, b-tub, MCM7 and RPB2 5–7 and 7–11 from 39 specimens. Support values (Likelihood BS) given when BS ≥ 60%. The scale bars indicate 0.03 substitution.

For the final concatenated dataset, we included 131 nucleotide sequences of mtSSU, b-tub, MCM7, and the two parts of RPB2 (5–7 and 7–11). This dataset encompassed 4140 nucleotide positions (843 bp for mtSSU, 708 bp for b-tub, 597 bp for MCM7, and 1074 bp for RPB2 5–7 and 918 bp for RPB2 7–11) with 39 terminals. It incorporates representatives from selected genera within the Collemataceae, in conjunction with additional data sourced from GenBank (utilized in Otálora et al 2013a, 2013b; Košuthová et al. 2019), with *Enchyliumbachmanianum* as the outgroup. None of the sequences of the “*Nigrescens*” and “*Flaccidum*”-groups previously used in phylogenetic analyses and uploaded to GenBank (Otálora et al. 2010, 2013a, 2013b; Miadlikowska et al. 2014), were included in this study due to concerns about specimen misidentification. Sequences used in this study are summarized in Fig. 1, Table 2.

## ﻿Results and discussion

### ﻿Dataset and phylogenetic analyses

We generated 104 new sequences (Table 2), comprising 28 mtSSU, 23 b-tub, 16 MCM7, 27 RPB2. Our analyses resulted in a topology (Fig. 1) very similar to the findings of Otálora et al. (2013a, 2013b).

Our phylogenetic analysis reveals that European *Collema* should be treated as including *C.curtisporum*, *C.flaccidum*, *C.furfuraceum*, *C.nigrescens*, *C.ryssoleum*, *C.subflaccidum* and *C.subnigrescens*, but excluding *C.glebulentum* as this species clearly groups within *Leptogium* together with *Leptogiumazureum*, the conserved type species of *Leptogium* (Fig. 1). The groups within *Collema* as informally circumscribed by Degelius (1954), are not supported by our phylogeny. The “*Flaccidum*”-group, consisting of *C.flaccidum* and *C.subflaccidum*, is nested within the “*Nigrescens*” -group. This indicates that these two informal groups are not useful for a phylogenetically based classification (Fig. 1). *Collemafurfuraceum* is further non-monophyletic, suggesting the need for further taxonomic revision.

### ﻿Morphological analyses

**Figure 2.**
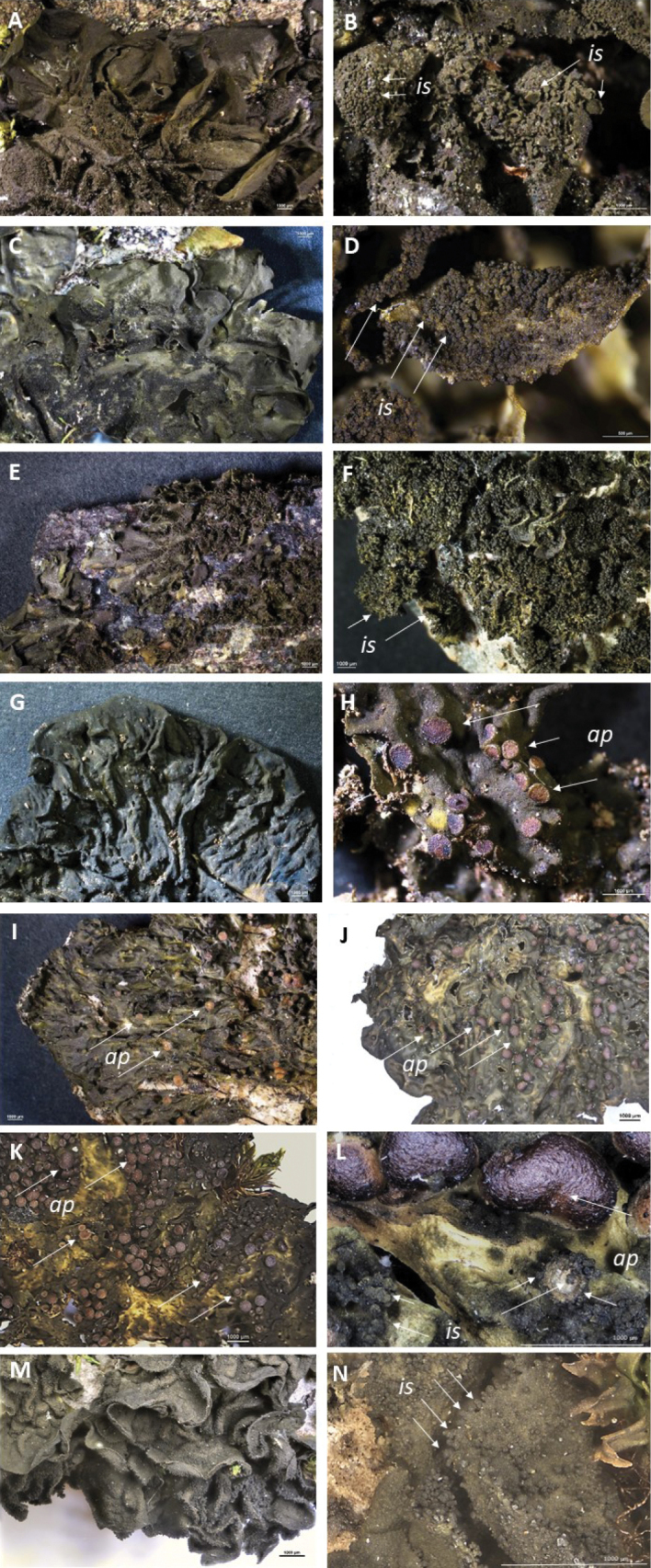
Thallus habitus and isidia of **A***Collemaflaccidum* (UPS-L872188) **B***C.flaccidum* (UPS-L872188) **C***C.subflaccidum* (S-F492349) **D***C.subflaccidum* (S-F492350) **E***Leptogiumglebulentum* (S-L49768) **F***L.glebulentum* – dwarf form (S-L49768) **G***C.ryssoleum* (UPS-L933969) **H***C.ryssoleum* (UPS-L933969) **I***C.subnigrescens* (S-F57732) **J***C.curtisporum* (UPS-L111603) **K***C.nigrescens* (UPS-L934034) **L***C.nigrescens* (UPS-L934034) **M***C.furfuraceum* (UPS-L934040) **N***C.furfuraceum* (UPS-L934040). ***is*** = isidia, ***ap*** = apothecium. Scale bar: **A–C**, **E–N**: 1 mm, **D**: 0.5 mm.

**Figure 3.**
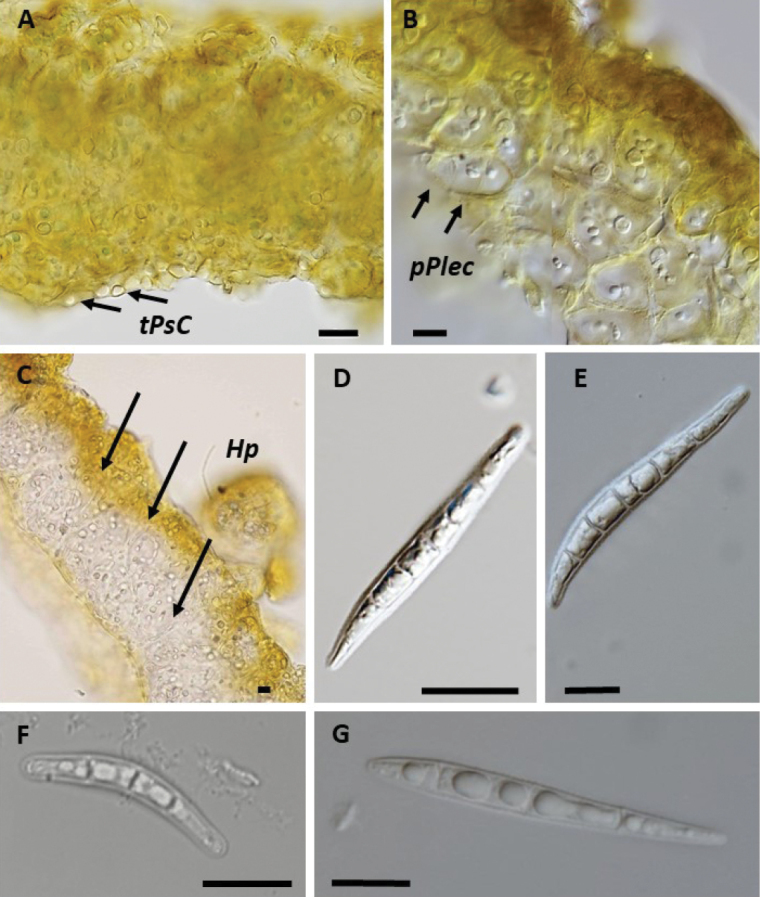
Thalli transversal cross-sections in water **A–C**, ascospores **D–G A** thallus with typical pseudocortex (*Leptogiumglebulentum* S-L49768) **B** thallus paraplectenchymateous throughout (*Leptogiumglebulentum* S-L49768), **C** thallus with hyphae which are perpendicular to the surface (*C.furfuraceum* S-F492354) **D** spore of *C.ryssoleum* (UPS-L178905) **E** spore of *C.subnigrescens* (GZU66-201) **F** spore of *C.curtisporum* (UPS-L111603) **G***C.nigrescens* (S-F492352), ***tPsC*** = typical pseudocortex, ***Hp*** = hyphae, ***pPlect*** = paraplechtenchyma. Scale bar: 10 μm.

Degelius (1954) differentiated his informal groups in European *Collema* based on morphological characteristics, noting that species within the “*Flaccidum*”-group are all isidiate but not pustulate (Table 1, Fig. 2A–F). Isidia in *Collemaflaccidum* are typically numerous and squamiform (flattened) when fully developed, rounded, and about 0.2–0.5 mm broad or larger. Larger isidia are often crenate or lobulate, with occasional teretiform isidia mixed in (Fig. 2B). In contrast, *C.subflaccidum* has smaller, globular isidia approximately 0.05–0.1 mm in diameter that become slightly teretiform as they mature, reaching at least 0.3 mm in length, with simple or slightly branched forms (Fig. 2C, D). *Collemaglebulentum* (Fig. 2E, F) has, sometimes together with a primitive pseudocortex, a distinct typical pseudocortex, often developed on the lower surface, especially on smaller lobes. When a typical pseudocortex is present, it is composed of several cell layers where the cells can be quite large, reaching up to 15 µm in diameter (Fig. 3A). Parts of the thallus may have an entirely paraplectenchymatous structure. This characteristic, noted by Degelius (1954), actually supports the original classification of *C.glebulentum* in *Leptogium* (Fig. 3B). In areas where the thallus is not entirely paraplectenchymatous, its structure resembles that of other species in the “*Flaccidum*” and “*Nigrescens*”-groups. The entire thallus in species from these groups is composed of hyphae that are either loosely or compactly interwoven, or arranged distinctly perpendicular to the upper and lower cortices throughout the thallus (Fig. 3C).

Species within the “*Nigrescens*”-group are characterized by their pustulate and ridged thalli (Fig. 2G–P) and can be divided into non-isidiate species and those that produce isidia. Among the non-isidiate species, *Collemaryssoleum* (Fig. 2G, H) is distinct due to its spore morphology. The spores are short, measuring up to 40 µm in length, similar to those in *C.curtisporum*. However, unlike others in the group, *C.ryssoleum* spores are relatively wide (up to 8.5 µm) with acute ends (Table 1). Additionally, this species exhibits a unique Mediterranean distribution in the temperate zone and is adapted to a saxicolous habitat (Fig. 3D). *Collemasubnigrescens* is characterized by an up to 20 cm large thallus (Fig. 2I) and by its narrowly fusiform to irregularly clavate spores with twisted, acute ends (5-celled, occasionally up to 12 cells; Fig. 3E). It closely resembles *C.curtisporum*, which, however, has a smaller, up to 4 cm wide thallus (Fig. 2J) and shorter, mostly 3-celled bacillariform spores with obtuse ends and a typical “curved-acute shape” (Fig. 3F). Spores in *C.nigrescens* differ from the other species in being acicular to bacillariform, longer and thinner than those in *C.subnigrescens* (usually around 60 µm long and less than 5 µm wide), 6–13 celled (Fig. 3G).

Among the isidiate species, *Collemanigrescens* is notable for its globular isidia (ca 0.2 mm in diameter; Fig. 2K, L), whereas *C.furfuraceum* has thinner, teretiform isidia (ca 0.05–0.15 mm wide) that become coralloid and reach up to 0.3 mm in length (Fig. 2M, N). Degelius (1974) later included *Collemaluzonense* Räs. from the Philippines as a variety of C.furfuraceum and the var.luzorense, was distinguished by its euparaplectenchymatous excipulum proprium and frequent presence of white-pruinose apothecia. Another distinguishing feature of var.luzorense is its thicker lobes, reaching up to 300 µm, compared to 100 µm in var. furfuraceum. The var.luzorense has not been reported from Europe and the thallus thickness of the samples (which were all sterile) in our study suggests that they belong in var.furfuraceum.

Given the results of our phylogenetic analysis, it is crucial to study *C.furfuraceum* further. This study is under way, whereby we also plan to investigate the potential role of isidia as a distinguishing feature for species identification.

## ﻿Conclusion

This study provides new insights into the phylogenetic relationships and morphological diversity among the European *Collema* species. Our results show that *C.glebulentum* belong in *Leptogiums. str.*, which is supported by its paraplectenchymatous thallus structure. Phylogenetic analysis reveals that Degelius´ (1954)*Collema* “*Flaccidum*” and “*Nigrescens*”-groups are not supported by the actual phylogenetic relationships. The study indicates that there are still substantial taxonomic issues worth studying and clarifying within European *Collema* s. str. We will present the results of such a study in a forthcoming publication.

## ﻿Additional information

### Author ORCIDs

Alica Košuthová https://orcid.org/0000-0001-5991-7444

Mats Wedin https://orcid.org/0000-0002-8295-5198

### Data availability

All of the data that support the findings of this study are available in the main text.
